# TSNAdb v2.0: The Updated Version of Tumor-specific Neoantigen Database

**DOI:** 10.1016/j.gpb.2022.09.012

**Published:** 2022-10-06

**Authors:** Jingcheng Wu, Wenfan Chen, Yuxuan Zhou, Ying Chi, Xiansheng Hua, Jian Wu, Xun Gu, Shuqing Chen, Zhan Zhou

**Affiliations:** 1 Institute of Drug Metabolism and Pharmaceutical Analysis and Zhejiang Provincial Key Laboratory of Anti-Cancer Drug Research, College of Pharmaceutical Sciences, Zhejiang University, Hangzhou 310058, China; 2 Alibaba-Zhejiang University Joint Research Center of Future Digital Healthcare, Alibaba DAMO Academy, Hangzhou 311121, China; 3 The Second Affiliated Hospital of School of Medicine, and School of Public Health, Zhejiang University, Hangzhou 310058, China; 4 Innovation Institute for Artificial Intelligence in Medicine, Zhejiang University, Hangzhou 310018, China; 5 Department of Genetics, Development and Cell Biology, Iowa State University, Ames, IA 50011, USA

**Keywords:** Neoantigen, Tumor immunotherapy, Human leukocyte antigen, Somatic mutation, Database

## Abstract

In recent years, **neoantigens** have been recognized as ideal targets for **tumor immunotherapy**. With the development of neoantigen-based tumor immunotherapy, comprehensive neoantigen **databases** are urgently needed to meet the growing demand for clinical studies. We have built the tumor-specific neoantigen database (TSNAdb) previously, which has attracted much attention. In this study, we provide TSNAdb v2.0, an updated version of the TSNAdb. TSNAdb v2.0 offers several new features, including (1) adopting more stringent criteria for neoantigen identification, (2) providing predicted neoantigens derived from three types of **somatic mutations**, and (3) collecting experimentally validated neoantigens and dividing them according to the experimental level. TSNAdb v2.0 is freely available at https://pgx.zju.edu.cn/tsnadb/.

## Introduction

Tumor neoantigens are tumor-specific antigens derived from somatic mutations in tumor cells, which have been recognized as ideal targets for tumor immunotherapy in recent years [1], [2], [3], [4]. Due to the huge workload for experimental verification, it is preferred to utilize cancer genomics and bioinformatics for neoantigen identification. Numerous prediction tools considering the biological process of neoantigen generation, such as human leukocyte antigen (HLA)–peptide binding [5], [6], [7], have been developed, which have been embedded in neoantigen prediction pipelines such as pVACtools [8], tumor-specific neoantigen detector (TSNAD) [9], [10], and pTuneos [11]. Neoantigen-related databases such as TRON cell line portal (TCLP) [12], the cancer immunome atlas (TCIA) [13], and tumor-specific neoantigen database (TSNAdb) [14] have also been created for better usage of neoantigens in clinical research. In TSNAdb v1.0, we took the complex of mutated peptides and HLA class I molecules (peptide–HLA pairs, pHLAs) as tumor neoantigens and predicted binding affinities between mutated/wild-type pHLAs by NetMHCpan v2.8/v4.0. We then obtained 3,707,562/1,146,961 potential neoantigens derived from single nucleotide variants (SNVs) of 7748 tumor samples from The Cancer Genome Atlas (TCGA, https://portal.gdc.cancer.gov/). With the development of neoantigen-based tumor immunotherapy, neoantigens from other types of mutations have been identified, and more experimental data have been generated [15], [16]. Therefore, it is urgent to perform system updates for the TSNAdb v1.0.

**Figure 1:**
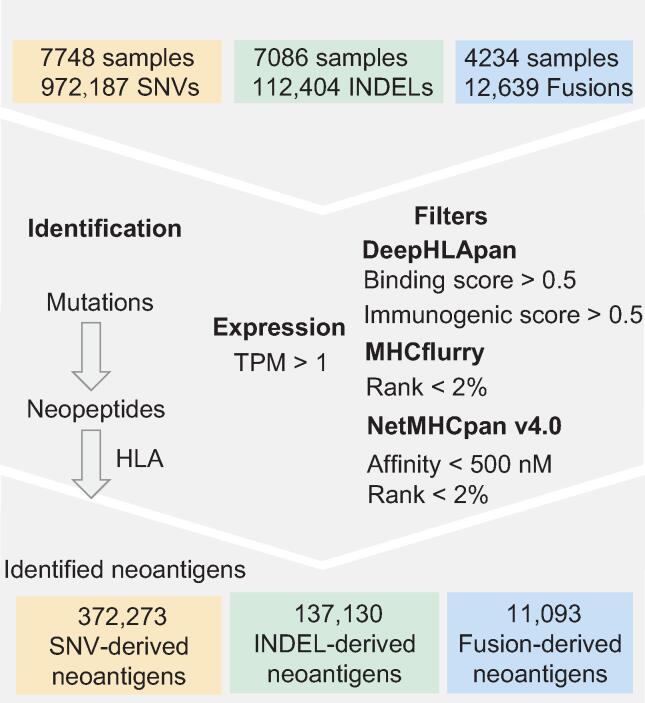
**The neoantigen prediction process of TSNAdb v2.0** SNV, single nucleotide variant; INDEL, insertion/deletion; Fusion, gene fusion; HLA, human leukocyte antigen; TPM, transcripts per million reads.

Here, we present an updated version of TSNAdb v1.0 that improves on the following points. (1) More stringent criteria were used for neoantigen identification to reduce the high false-positive rate of neoantigen prediction in practice. Only the pHLAs that met the thresholds of three tools were considered potential neoantigens (Figure 1). The pHLAs would not be considered neoantigens if the mutated genes were not expressed in the tumor cells. (2) We provided predicted neoantigens derived not only from SNVs but also from insertions/deletions (INDELs) and gene fusions (Fusions). In total, 372,273 SNV-derived neoantigens, 137,130 INDEL-derived neoantigens, and 11,093 Fusion-derived neoantigens were obtained. The mean number of neoantigens generated for each SNV (0.38) was lower than each INDEL (1.22) or Fusion (0.88). (3) We collected as many experimentally validated neoantigens from public databases and literature as possible (1856 neoantigens) and divided them into three tiers according to the level of experimental verification. Corresponding genes and mutations of the collected neoantigens were linked to the cancer-driving site profiling database (CandrisDB) [17] since neoantigens derived from driver genes or driver mutations would be ideal targets for tumor immunotherapy [18].

We believe that the updated database will contribute to neoantigen-based tumor immunotherapy and that the database will continue updating in the aspect of predicting neoantigens from more types of mutations and collecting more experimentally validated neoantigens.

## Database content and usage

### Data collection and preprocessing

The SNVs, INDELs, and the expression level of corresponding genes were collected from TCGA. Mutated nucleotide sequences generated by SNVs and INDELs are translated into mutated amino acid sequences and have been decomposed into 8 to 11 peptides using the pipeline TSNAD v2.0 [10]. The Fusions were collected from Gao et al. [19], and the mutated proteins were generated by STAR-Fusion [20]. The HLA alleles of corresponding samples were collected from TCIA. Finally, 972,187 SNVs from 7748 samples, 112,404 INDELs from 7086 samples, and 12,639 Fusions from 4234 samples were used for neoantigen prediction.

### Stricter criteria for neoantigen identification

Neoantigen-based tumor immunotherapy has shown good application prospects in clinical practice. However, the high false-positive rate of neoantigen prediction limits its usage. How to select high-confidence immunogenic neoantigens remains to be resolved. To reduce the potential false-positive rate in our predicted results, three tools (DeepHLApan, MHCflurry, and NetMHCpan v4.0) were used for neoantigen prediction, and only the pHLAs that met all the criteria of the three tools were considered potential neoantigens (Figure 1). The reason we chose these three tools is as follows: NetMHCpan [7] is the most frequently used tool for neoantigen prediction in clinical practice. MHCflurry [6] obtains the prediction neoantigen efficiently and with high quality. DeepHLApan [5] considers both HLA-peptide binding and immunogenicity of pHLA that the other two tools have not taken into consideration for high-confidence neoantigen prediction. The threshold of each tool is as follows. For NetMHCpan v4.0, pHLA with rank < 2% or affinity < 500 nM is considered binding, and we used both thresholds to select higher quality neoantigens. The output of MHCflurry is rank % and has no specific threshold. We set rank < 2% as the threshold, which is the same as NetMHCpan v4.0. The predicted scores of DeepHLApan are posterior probabilities, so we set the threshold to 0.5. In addition, the pHLAs whose corresponding genes were not expressed [transcripts per million reads (TPM) < 1] were removed.

### Neoantigens derived from three types of mutation

**Figure 2:**
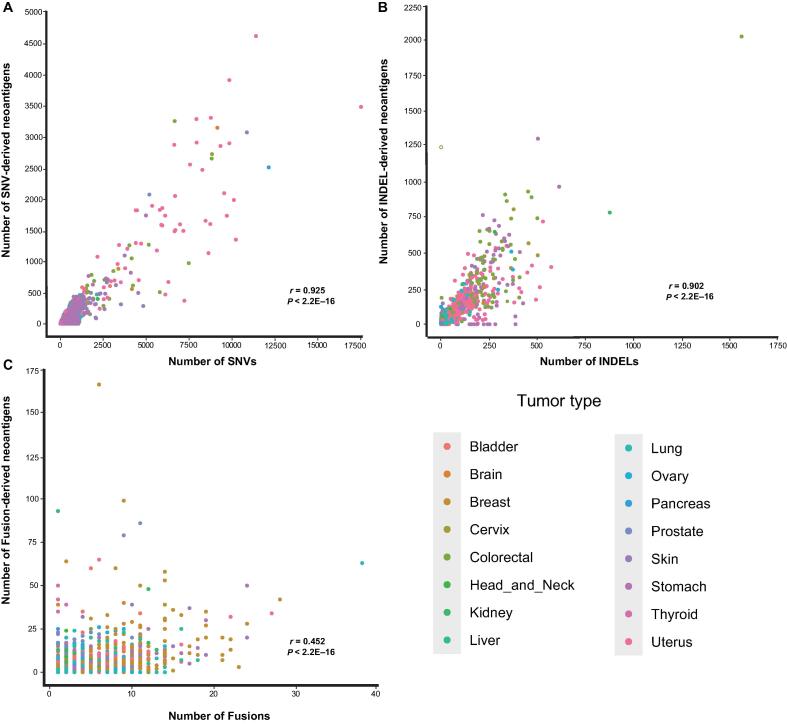
**The relationship between the number of mutations and the number of neoantigens across 16 tumor types of three mutation types** **A.** The relationship between the number of SNVs and SNV-derived neoantigens. **B.** The relationship between the number of INDELs and INDEL-derived neoantigens. **C.** The relationship between the number of Fusions and Fusion-derived neoantigens. The Pearson correlation coefficient is used for evaluation.

Neoantigens are not only generated from SNVs but also generated from other mutations, such as INDELs and Fusions. Based on the analysis of different types of mutations in TCGA tumor samples, we provided 137,130 INDEL-derived neoantigens and 11,093 Fusion-derived neoantigens into TSNAdb v2.0. The number of predicted neoantigens derived from SNVs was greater than that derived from INDELs and Fusions due to the greatest number of SNVs among the somatic mutations collected. However, the average number of neoantigens derived from each SNV (0.38) was less than that derived from each INDEL (1.22) or each Fusion (0.88) (Table 1). We further explored the relationship between the number of mutations and neoantigens for the three mutation types. The results showed that the numbers of SNV-derived neoantigens and INDEL-derived neoantigens had positive correlations with the numbers of SNVs and INDELs, with the Pearson correlation coefficient *r* = 0.925 and *r* = 0.902, respectively (Figure 2A and B). There was no significant correlation between the number of Fusion-derived neoantigens and the number of Fusions (Figure 2C, *r* = 0.452), which might be attributed to the fact that the number of neopeptides each Fusion generated varies greatly.

**Table 1:** The distribution of mutations and neoantigens across 16 tumor types

**Mutation type**	**Tissue**	**No. of samples**	**No. of mutations**	**No. of neoantigens**	**No. of neoantigens per mutation**
SNV	Bladder	408	74,707	21,070	0.38
	Brain	151	16,197	5227	
	Breast	982	62,211	18,238	
	Cervix	286	46,607	11,906	
	Colorectal	531	165,293	39,116	
	Head and neck	495	55,968	13,728	
	Kidney	681	26,170	8499	
	Liver	358	28,024	5987	
	Lung	999	212,861	52,801	
	Ovary	305	28,478	7631	
	Pancreas	166	17,221	3985	
	Prostate	493	16,424	5363	
	Skin	466	206,907	43,882	
	Stomach	411	103,103	24,497	
	Thyroid	488	4425	1218	
	Uterus	528	415,499	109,125	

INDEL	Bladder	389	2890	3532	1.22
	Brain	130	366	375	
	Breast	888	5207	6123	
	Cervix	257	3095	4234	
	Colorectal	520	21,327	29,876	
	Head and neck	478	3569	4913	
	Kidney	648	5318	6395	
	Liver	353	2120	2143	
	Lung	983	10,790	12,407	
	Ovary	296	4260	4441	
	Pancreas	141	457	806	
	Prostate	416	1448	2119	
	Skin	430	1965	2557	
	Stomach	393	19,912	24,838	
	Thyroid	248	1746	1657	
	Uterus	516	27,934	30,714	

Fusion	Bladder	269	809	819	0.88
	Brain	92	244	245	
	Breast	787	3390	3457	
	Cervix	139	280	215	
	Colorectal	167	328	231	
	Head and neck	285	532	530	
	Kidney	171	318	420	
	Liver	179	513	423	
	Lung	716	1992	710	
	Ovary	316	983	609	
	Pancreas	72	130	161	
	Prostate	381	1079	1120	
	Skin	287	841	918	
	Stomach	178	559	486	
	Thyroid	110	140	250	
	Uterus	85	501	499	

*Note*: SNV, single nucleotide variant; INDEL, insertion/deletion; Fusion, gene fusion.

### Shared neoantigens generated from frequent somatic mutations

Currently, most neoantigen-targeted immunotherapies are personalized and expensive, which led us to wonder if we could identify shared neoantigens that can be applied to a wider range of tumor patients. Here, we analyzed the frequency of each neoantigen and obtained 16,913 neoantigens shared in at least two tumor samples (Table S1). Among three SNV-derived neoantigens shared in more than 20 samples, the mutated peptides are generated from *BRAF* and *KRAS*, which are well-known cancer driver genes. The most frequent shared neoantigen derived from SNV is the complex of HLA-B57:01 and mutated peptide GLAT*E*KSRW generated by *BRAF* V600E, which is present in 41 tumor samples. The complex of HLA-A02:01 and the neopeptides RLMAPVGSV and SLLTQPSPA generated by the frameshift mutation *XYLT2* G529Afs*78 are the most frequent neoantigens among INDEL-derived neoantigens, which both appear in 31 samples (Table 2). The two Fusion-derived shared neoantigens are the complex of HLA-A02:01 and the neopeptides ALNS*EALSVV* and ALNS*EALSV* generated by the fusion of the *TMPRSS2* and *ERG* genes*,* which both appear in 14 samples (Table S1). We believe that these shared neoantigens are expected to be ideal drug targets for tumor immunotherapy, which might need further experimental validation.

**Table 2:** The detailed information of shared neoantigens present in more than 20 samples

**Mutation type**	**Gene**	**Mutation**	**HLA**	**Peptide**	**No. of samples**
SNV	*BRAF*	V600E	HLA-B57:01	GLATEKSRW	41
SNV	*KRAS*	G12D	HLA-B08:01	DGVGKSAL	37
INDEL	*XYLT2*	G529Afs*78	HLA-A02:01	RLMAPVGSV	31
INDEL	*XYLT2*	G529Afs*78	HLA-A02:01	SLLTQPSPA	31
SNV	*KRAS*	G12V	HLA-A03:01	VVGAVGVGK	30
INDEL	*SETD1B*	H8Tfs*27	HLA-C07:01	LRARGGTTI	29
INDEL	*PLEKHA6*	V328Yfs*172	HLA-A02:01	IMMSWMPPL	25
INDEL	*PLEKHA6*	V328Yfs*172	HLA-A02:01	VLSGCHLAV	25
INDEL	*XYLT2*	G529Afs*78	HLA-C07:02	GRTPTTRLM	25
INDEL	*PLEKHA6*	V328Yfs*172	HLA-A02:01	FTPLSAHPV	25
INDEL	*PLEKHA6*	V328Yfs*172	HLA-A02:01	CLAGSLSTM	25
INDEL	*PLEKHA6*	V328Yfs*172	HLA-A02:01	SAMPSAMGV	25
INDEL	*PLEKHA6*	V328Yfs*172	HLA-A02:01	SIMMSWMPPL	25
INDEL	*XYLT2*	G529Afs*78	HLA-B07:02	RPACTCISM	23
INDEL	*XYLT2*	G529Afs*78	HLA-B07:02	HPQWAPHSA	23
INDEL	*XYLT2*	G529Afs*78	HLA-B07:02	RPTGRTPTTRL	23
INDEL	*XYLT2*	G529Afs*78	HLA-B07:02	SPGACRPAC	23
INDEL	*XYLT2*	G529Afs*78	HLA-B07:02	RPTGRTPTT	23
INDEL	*MUC4*	L1937Pfs*1069	HLA-B07:02	HPQVTPPL	23
INDEL	*MUC4*	L1937Pfs*1069	HLA-B07:02	HPQVTPPLF	23
INDEL	*SETD1B*	H8Tfs*27	HLA-C07:02	LRARGGTTI	22
INDEL	*MUC4*	L1937Pfs*1069	HLA-B07:02	LPQHPQVTPPL	21

*Note*: fs*78 indicates that 78 amino acids have been changed after the frameshift site. HLA, human leukocyte antigen; fs, frameshift.

### Experimentally validated neoantigens

On the “Validation” page of TSNAdb v1.0, we only collected experimental data about wild-type pHLAs that were difficult to identify as neoantigens due to the limited binding data between mutated pHLAs. With the development of clinical studies on neoantigen-based tumor immunotherapy, a large number of experimental results have provided a rich source for the functional confirmation of neoantigens. Here, we collected experimentally validated mutated pHLAs not only from several neoantigen databases (dbPepNeo [21], NeoPeptide [22], NEPdb [23], and Cancer Antigenic Peptide Database [24]) but also from published literature through data mining. For the neoantigens without gene or mutation information, BLAST was used to determine the mutated genes and the positions of somatic mutations at proteins. All collected data were further checked to determine whether the neoantigens were immunogenic or presented to the cell surface, and the collected neoantigens were divided into three tiers according to the experimental level. Neoantigens that have been both validated as immunogenic and to be presented to the cell surface were labeled tier 1, while those only validated as immunogenic were labeled tier 2, and those only validated to be presented to the cell surface were labeled tier 3. We collected 1856 experimental neoantigens, among which 67 neoantigens were classified as tier 1, 1190 neoantigens were classified as tier 2, and 599 neoantigens were classified as tier 3. Among the collected neoantigens, most were SNV-derived (22 were Fusion-derived, 125 were INDEL-derived, 23 were noncoding-derived, 33 were RNA splice-derived, and the remaining were SNV-derived) and enriched in several tumor types (430 belonged to lung cancer, 477 belonged to skin cancer, 361 belonged to B-cell lymphoma, and 123 belonged to colorectal cancer).

### The usage of TSNAdb v2.0

The web interface of TSNAdb v2.0 contains seven pages: “Home”, “Browse”, “Search”, “Collected”, “Tools”, “Download”, and “Help”. The pages “Home”, “Download”, and “Help” are similar to those presented in TSNAdb v1.0. The “Tools” page is newly added to provide the links of DeepHLApan and TSNAD, which we developed previously for neoantigen prediction. Major changes (such as more presentation forms, more correlation analysis, and more meaningful links) have been made in the pages “Browse”, “Search”, and “Collected” compared to the TSNAdb v1.0.

**Figure 3:**
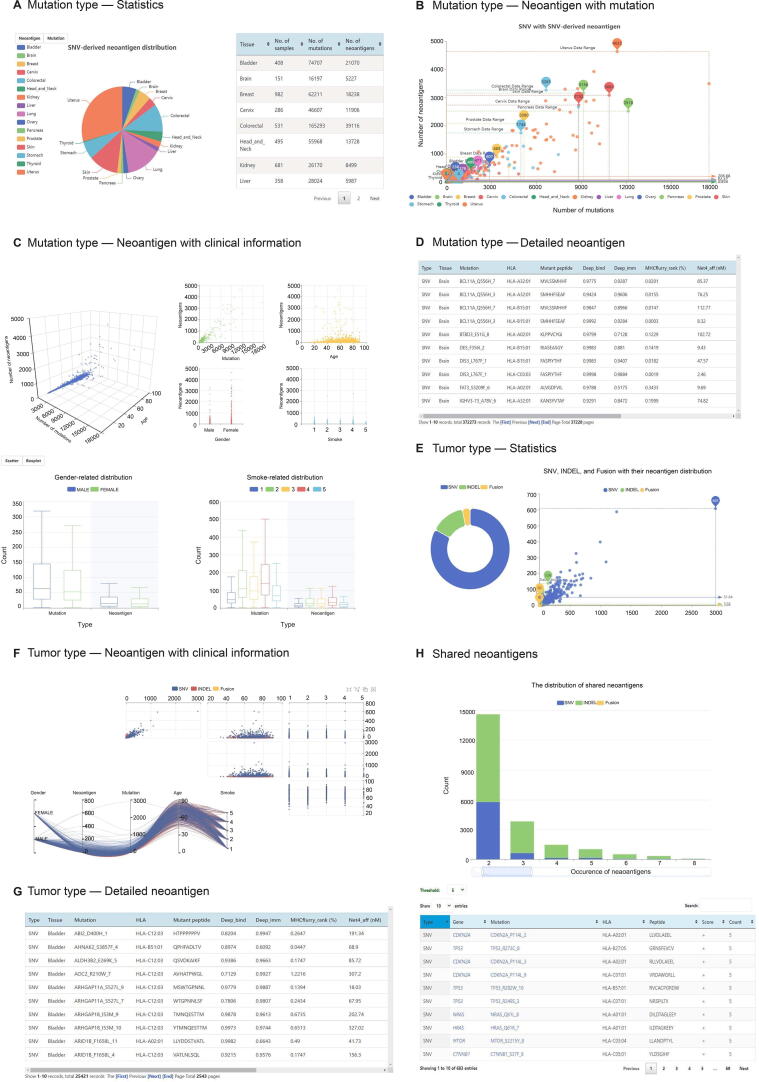
**Screenshots of the “Browse” page of TSNAdb v2.0** **A.** The “Statistics” part of the “Mutation type” page. **B.** The “Neoantigen with mutation” part of the “Mutation type” page. **C.** The “Neoantigen with clinical information” part of the “Mutation type” page. **D.** The “Detailed neoantigen” part of the “Mutation type” page. **E.** The “Statistics” part of the “Tumor type” page. **F.** The “Neoantigen with mutation” part of the “Tumor type” page. **G.** The “Detailed neoantigen” part of the “Tumor type” page. **H.** The “Shared neoantigens” page.

On the “Browse” page, three subpages (“Mutation type”, “Tumor type”, and “Shared neoantigens”) are provided for customized neoantigen browsing. In “Mutation type”, four parts, “Statistics” (Figure 3A), “Neoantigen with mutation” (Figure 3B), “Neoantigen with clinical information” (Figure 3C), and “Detailed neoantigen” (Figure 3D), are displayed. In “Tumor type”, three parts, including the “Statistics” (Figure 3E), “Neoantigen with clinical information” (Figure 3F), and “Detailed neoantigen” (Figure 3G), are displayed. In the “Shared neoantigens”, the distribution of shared neoantigens that occur in at least two tumor samples is displayed. The table below the boxplot displays the shared neoantigens that meet different thresholds. The genes and mutations are linked to CandrisDB [17] to check whether they are driver genes or driver mutations since shared neoantigens derived from driver mutations would be potential ideal targets for tumor immunotherapy (Figure 3H).

The “Search” page contains the main page and two subpages “Gene” and “HLA”. On the main page of “Search”, users could search for desired neoantigens by selecting the mutation type, tumor type, and gene. Compared with the “Detailed neoantigen” of the “Browse” page, it could provide more customized functions, such as sorting and searching. In the subpages “Gene” and “HLA”, the detailed neoantigens and their distribution of selected genes or HLAs would be displayed once searching. The displayed pie charts are linked with the bellowed table that the detailed neoantigens would be changed once clicking on the part of the pie charts.

On the “Collected” page, all collected neoantigens are validated by experiments to be presented to the cell surface or immunogenic, which are different from those in TSNAdb v1.0. The corresponding genes and mutations of neoantigens are linked to CandrisDB as those in the “Shared neoantigens”.

## Discussion and perspectives

Neoantigens play an important role in tumor immunotherapy. A comprehensive and high-confidence neoantigen database would greatly meet the needs of clinical research. In TSNAdb v2.0, we predict more mutation type-derived neoantigens with stricter criteria, present the tissue-specific and gene-specific distribution of candidate tumor-specific neoantigens of TCGA tumor samples, and collect 1856 neoantigens that have been experimentally validated, which is the most systematic database of tumor-specific neoantigens at present. Compared with other databases, TSNAdb v2.0 has several advantages as follows. First, TSNAdb v2.0 provides both high-quality predicted neoantigens and experimentally validated neoantigens, while most of the other databases except NEPdb only provide one of them. Compared with NEPdb, TSNAdb v2.0 provides more sources of predicted neoantigens and has richer forms of presentation. Second, TSNAdb v2.0 provides the analysis of shared neoantigens and links corresponding genes and mutations to CandrisDB to identify high-quality neoantigens, which other databases do not provide. Finally, TNSAdb will be updated continuously to provide constant service for related researchers and clinicians. We believe that it would certainly contribute to neoantigen-based tumor immunotherapy.

However, neoantigens are derived not only from SNVs, INDELs, and Fusions but also from splice variants [15], the mitochondrial genome [16], and translated unannotated open reading frames [25]. It is necessary to predict all sources of neoantigens to construct a comprehensive neoantigen database. Limited by the difficulty of collecting other mutations and corresponding HLAs, we only chose three sources of neoantigen in this version of the database. In the following update of TNSAdb, we would add neoantigens from more sources and collect more validated neoantigens to construct a more comprehensive neoantigen database.

## Data availability

TSNAdb v2.0 is freely available at https://pgx.zju.edu.cn/tsnadb/.

## Competing interests

The authors have declared no competing interests.

## CRediT authorship contribution statement

**Jingcheng Wu:** Methodology, Formal analysis, Writing – original draft, Visualization. **Wenfan Chen:** Formal analysis, Investigation. **Yuxuan Zhou:** Data curation, Investigation. **Ying Chi:** Methodology, Resources. **Xiansheng Hua:** Resources. **Jian Wu:** Methodology. **Xun Gu:** Methodology. **Shuqing Chen:** Conceptualization, Resources. **Zhan Zhou:** Conceptualization, Supervision, Writing – review & editing. All authors have read and approved the final manuscript.

## Supplementary material

Supplementary Table S1 **The detailed information of neoantigens shared in at least two samples**
